# Diagnosis and management of interstitial lung disease

**DOI:** 10.1186/2213-0802-2-4

**Published:** 2014-02-13

**Authors:** Keith C Meyer

**Affiliations:** Department of Medicine, University of Wisconsin School of Medicine and Public Health, Madison, Wisconsin USA

**Keywords:** Interstitial lung disease, Idiopathic pulmonary fibrosis, Therapeutics, Diagnosis

## Abstract

The complex tasks of making a confident diagnosis of a specific form of interstitial lung disease (ILD) and formulating a patient-centered, personalized management plan in an attempt to achieve remission or stabilization of the disease process can pose formidable challenges to clinicians. When patients are evaluated for suspected ILD, an accurate diagnosis of the specific form of ILD that a patient has developed must be made to provide the patient with useful prognostic information and to formulate an appropriate management plan that can relieve symptoms and restore or significantly improve quality of life. A well-performed patient history and physical examination provides invaluable information that can be combined with appropriate laboratory testing, imaging, and, if needed, tissue biopsy to reach a confident ILD diagnosis, and high-resolution computed tomography (HRCT) of the thorax is usually a key component of the diagnostic evaluation. If treatment is indicated, many forms of ILD can respond significantly to immunosuppressive anti-inflammatory therapies. However, ILD accompanied by extensive fibrosis may be difficult to treat, and the identification of an effective pharmacologic therapy for idiopathic pulmonary fibrosis (IPF) has remained elusive despite the completion of many phase 3 clinical trials over the past decade. Nonetheless, patients with IPF or advanced forms of non-IPF ILD can benefit significantly from detection and treatment of various co-morbid conditions that are often found in patients (especially the elderly patient), and supportive care (oxygen therapy, pulmonary rehabilitation) can have a beneficial impact on quality of life and symptom palliation. Finally, lung transplantation is an option for patients with progressive, advanced disease that does not respond to other therapies, but only a relatively small subset of patients with end-stage ILD are able to meet wait listing requirements and eventually undergo successful lung transplantation.

## Introduction

Well over one hundred different forms of interstitial lung disease (ILD) have been described (see Table 1 for major categories). These diffuse infiltrative lung disorders are typically characterized by the presence of inflammation and altered lung interstitium, and specific forms of ILD can be differentiated from one another when clinical data, radiologic imaging, and pathologic findings (if lung biopsy is needed) are combined to reach a confident diagnosis [1, 2]. The histopathologic changes in the lungs of patients with ILD can range from granulomatous inflammation without parenchymal fibrosis in patients with sarcoidosis to extensive pulmonary fibrosis with architectural distortion of the lung in patients with idiopathic pulmonary fibrosis (IPF). Some forms of ILD have been linked to specific genetic abnormalities (e.g. Hermansky-Pudlak syndrome, familial pulmonary fibrosis), and a number of gene variants have been associated with an increased risk to develop ILD disorders such as IPF, sarcoidosis, or chronic beryllium disease (CBD). Interstitial lung disease can also complicate connective tissue disorders (CTD), and lung histopathologic changes can have features of usual interstitial pneumonia (UIP) or non-specific interstitial pneumonia (NSIP) patterns in CTD-associated ILD [3].

**Table 1:** **Interstitial lung disorders: major categories**

• Idiopathic interstitial pneumonia	• Sarcoidosis
o Idiopathic pulmonary fibrosis (IPF)	• Hypersensitivity pneumonitis
o Non-specific interstitial pneumonia (NSIP)	• Iatrogenic pneumonitis/fibrosis (drug-induced ILD, radiation injury)
o Cryptogenic organizing pneumonia (COP)	• Eosinophilic ILD (e.g. eosinophilic pneumonia)
o Respiratory bronchiolitis interstitial lung disease (RBILD)	• Occupational lung disease
o Desquamative interstitial pneumonia (DIP)	• Inherited disorders (e.g. familial pulmonary fibrosis, Hermansky-Pudlak syndrome)
o Acute interstitial pneumonia (AIP)	• Primary disorders (e.g. pulmonary Langerhans cell histiocytosis)
o Lymphoid interstitial pneumonia (LIP)	
• Connective tissue disease-associated interstitial lung disease (CTD-ILD)	

Successful management of patients with ILD is dependent upon establishing an accurate and specific diagnosis [1, 2]. Because various forms of ILD such as IPF, non-IPF forms of idiopathic interstitial pneumonia (IIP), CTD-ILD, and hypersensitivity pneumonitis (HP) can have similar clinical presentations, patients with suspected ILD must undergo an evaluation that adequately establishes a confident diagnosis of a specific ILD, as treatment and various management decisions are diagnosis-specific and may vary considerably according to the specific form of ILD that is diagnosed. This manuscript will focus on [1] the appropriate steps that are required to make an accurate diagnosis of specific types of ILD, [2] general approaches to disease monitoring and management, and (3) therapies for specific disorders such as IPF.

## Review

### Clinical evaluation

A thorough and comprehensive history may provide invaluable information that can suggest certain entities and provide suspicion that a patient may have a specific diagnosis such as hypersensitivity pneumonitis (HP) or CTD-ILD (Table 2). Additional clues to an ultimate diagnosis can be provided by pulmonary and extra-pulmonary physical examination findings (Table 3), and the differential diagnosis can be considerably narrowed when key elements of the patient interview and physical examination findings are combined with appropriate measurements of lung function, specific blood tests (Table 4) such as autoimmune serologies to assist in the detection of CTD if such are indicated, extra-pulmonary tissue sampling (e.g. lymph node or skin biopsy), and thoracic imaging.

**Table 2:** **Clues from the initial evaluation that suggest specific types of ILD**

History elicited	Frequently associated ILD or complications of ILD
Rapid onset and worsening	AIP
	Infection
	Acute HP, acute EP
	Drug reaction
	COP
	CTD (e.g. acute lupus pneumonitis)
	DAH (e.g. GPS)
Smoking	RB-ILD, DIP, PLCH
Occupation: Pipefitter, foundry worker, coal miner,	Pneumoconiosis
Pneumotoxic drug exposure	Drug-induced ILD
Hemoptysis	DAH, pulmonary capillaritis, pulmonary venoocclusive disease, LAM
	Superimposed complications (e.g. pulmonary emboli, lung neoplasm)
Pleurisy	CTD (SLE, RA)
Wheezing	HP, EP
Eye symptoms	CTD, sarcoidosis, PAG
Impaired vision combined with albinism & Puerto Rican heritage	HPS
Rash	Sarcoidosis, CTD
Exposure to organic antigens at home or at work (e.g. birds, grain dust, humidifiers, visible molds, hot tubs,etc.)	HP
	Occupational ILD
Abnormal GER, GERD, dysphagia	CTD (especially scleroderma), IPF
Sicca symptoms	Sjögren’s disease
Raynaud’s phenomenon	CTD
Arthralgias, arthritis	CTD, sarcoidosis
Myalgias, muscle weakness	DM-PM
Morning stiffness	RA, CTD
Age >70 years	IPF > other ILD if HRCT suspicious for IIP

AIP = acute interstitial pneumonia; COP = cryptogenic organizing pneumonia; CTD = connective tissue disease; DAH = diffuse alveolar hemorrhage; DM-PM = dermatopolymyositis; EP = eosinophilic pneumonia; GER = gastroesophageal reflux; GERD = gastroesophageal reflux disease; GPS = Goodpasture’s syndrome; HP = hypersensitivity pneumonitis; HPS = Hermansky-Pudlak syndrome; IIP = idiopathic interstitial pneumonia; IPF = idiopathic pulmonary fibrosis; LAM = lymphangioleiomyomatosis; PAG = polyangiitis with granulomatosis; PLCH = pulmonary Langerhans cell histiocytosis; SLE = systemic lupus erythematosus; RA = rheumatoid arthritis.

Reprinted with permission from *Interstitial Lung Disease: A Practical Approach*. Meyer KC, Raghu G: **Patient evaluation**. Second Edition, New York: Springer; 2011:3–16.

**Table 3:** **Clues from the physical examination and their disease associations**

Organ system	Finding	Associated ILD or its complications
Lung	Velcro crackles	IPF, asbestosis > > other
	Squeaks	HP, bronchiolitis
	Pleural rub	RA, SLE
Skin	Erythema nodosum	Sarcoidosis, CTD, Behçet’s disease
	Maculopapular rash	CVD, drugs, sarcoidosis, amyloid
	Heliotrope rash	DM-PM
	Gottron’s papules	DM-PM
	Café-au-lait spots	Neurofibromatosis
	Albinism	HPS
	Telangiectasia	Scleroderma
	Calcinosis	Scleroderma, DM-PM
	Subcutaneous nodules	RA, neurofibromatosis, vasculitis
	Cutaneous vasculitis	PAG, RA, MPA, SLE, drug reaction
	Mechanic’s hands	DM-PM
	Tight skin/ulcerations over digits	Scleroderma
Eyes	Uveitis	Sarcoidosis, Behçet’s disease, AS
	Scleritis	SLE, scleroderma, sarcoidosis, PAG
	Keratoconjunctivitis sicca	Sjögren’s disease, CTD
	Lacrimal gland enlargement	Sarcoidosis
	Horizontal nystagmus	HPS
Salivary glands	Enlarged	Sjögren’s disease, sarcoidosis
Lymphatic	Lymphadenopathy	Sarcoidosis, lymphangitic CA, lymphoma
Reticuloendothelial	Hepatosplenomegaly	Sarcoidosis, LIP, CTD, EG, amyloid, lymphoma
Musculoskeletal	Muscle weakness, myositis	CTD (especially DM-PM), sarcoidosis
	Synovitis, arthritis	CTD, sarcoidosis
Nervous system	Neurologic dysfunction	Sarcoidosis, lymphangitic CA, NF, TS, CTD, PAG, MPA
Cardiovascular	Systemic hypertension	CTD, GPS, PAG, MPA, NF
	Prominent P2	Suggests secondary PH (IPF, CTD, end stage sarcoidosis)
	Pericardial rub	Sarcoidosis, SLE
Extremities	Digital clubbing	IPF, asbestosis > chronic HP, DIP > other fibrotic ILD
	Raynaud’s phenomenon	CTD

AS = ankylosing spondylitis; CA = cancer; CTD = connective tissue disease; DAH = diffuse alveolar hemorrhage; DM-PM = dermatopolymyositis; GPS = Goodpasture’s syndrome; HP = hypersensitivity pneumonitis; HPS = Hermansky-Pudlak syndrome; LAM = lymphangioleiomyomatosis; LCH = Langerhans cell histiocytosis; LIP = lymphoid interstitial pneumonia; MPA = microscopic polyangiitis; NF = neurofibromatosis; P2 = auscultated pulmonic valve closure sound; PAG = polyangiitis with granulomatosis; PH = pulmonary hypertension; RA = rheumatoid arthritis; SLE = systemic lupus erythematosus; TS = tuberous sclerosis.

Reprinted with permission from *Interstitial Lung Disease: A Practical Approach*. Meyer KC, Raghu G: **Patient evaluation**. Second Edition, New York: Springer; 2011:3–16.

**Table 4:** **Clues for specific diagnoses from blood and urine testing**

Laboratory test	Abnormal result	Suggested disorder
CBC	Microcytic anemia	Occult pulmonary hemorrhage
	Normocytic anemia	CTD, chronic disease
	Leukocytosis	Infection, hematologic malignancy
	Eosinophilia	Eosinophilic pneumonia, drug toxicity
	Thrombocytopenia	CTD, sarcoidosis
Calcium	Hypercalcemia	Sarcoidosis
Creatinine	↑	CTD, pulmonary-renal syndrome, sarcoidosis; amyloidosis
Liver function	↑ GGT, ALT, AST	Sarcoidosis, amyloidosis, CTD (polymyositis)
Urine	Abnormal sediment with RBC casts and/or dysmorphic RBCs	Vasculitis (CTD, PAG, GPS, MPA)
Muscle enzymes	↑Increased CK, aldolase	PM, DM-PM
Angiotensin Converting Enzyme (ACE)	↑	Sarcoidosis (non-specific; can be increased in other ILD)
Lymphocyte proliferation	Stimulated by beryllium	CBD
Serum antibodies	↓ Quantitative immunoglobulins	Immunodeficiency (CVID)
	↑ ANA, RF, anti-CCP	CTD, RA
	↑ C-ANCA	PAG
	↑ P-ANCA	CTD, vasculitis
	↑ anti-GBM	GPS
	Positive specific precipitin	Supportive of HP
	↑ anti-Jo-1 or other anti-synthetase autoantibodies	PM, DM-PM
	↑ SS-A, SS-B	Sjögren’s syndrome

CBD = chronic beryllium diseases; COP = cryptogenic organizing pneumonia; CTD = connective tissue disease; CVID = common variable immunodeficiency; DAH = diffuse alveolar hemorrhage; DM-PM = dermatopolymyositis; DIP = desquamative interstitial pneumonia; GPS = Goodpasture’s syndrome; HP = hypersensitivity pneumonitis; MPA = microscopic polyangiitis; PM = polymyositis; PAG = polyangiitis with granulomatosis; RA = rheumatoid arthritis;

Reprinted with permission from *Interstitial Lung Disease: A Practical Approach*. Meyer KC, Raghu G: **Patient evaluation**. Second Edition, New York: Springer; 2011:3–16.

The routine postero-anterior chest X-ray (CXR) can be highly suggestive of specific ILD entities (Table 5), and previous CXRs, if available, should be sought to determine whether the disease is acute versus chronic. Other studies should not be overlooked; as specific examples, abdominal computed tomographic (CT) scans usually show a substantial portion of the lower lung regions and thorax, and cervical spine CT scans can show extensive areas of the upper chest and lungs.

**Table 5:** **Thoracic imaging patterns**

Imaging modality	Pattern	Consistent ILD diagnoses, mimics of ILD, and/or complications of ILD
Routine CXR	Hilar lymphadenopathy	Sarcoidosis, silicosis, CBD, infection, malignancy
	Septal thickening	CHF, malignancy, infection, PVOD
	Lower lung zone predominance	IPF, asbestosis, DIP, CTD, NSIP
	Mid/upper lung zone predominance	Sarcoidosis, silicosis, acute HP, LCH, CBD, AS, chronic EP
	Peripheral lung zone predominance	COP, chronic EP, IPF
	Honeycomb change	IPF, asbestosis, chronic HP, sarcoidosis, fibrotic NSIP, CTD
	Small nodules	Sarcoidosis, HP, infection
	Cavitating nodules	PAG, mycobacterial infection, CA
	Migratory or fluctuating opacities	HP, COP, DIP
	Pneumothorax	PLCH, LAM, neurofibromatosis, TS
	Pleural involvement	Asbestosis, CTD, acute HP, malignancy, sarcoidosis, Radiation fibrosis
	Kerley B line prominence	Lymphangitic carcinomatosis, CHF
HRCT	Nodules	Sarcoidosis HP, CBD, pneumoconiosis, RA, malignancy
	Septal thickening	Edema, malignancy, infection, drug toxicity, PVOD
	Cyst formation	LAM, LCH, LIP, DIP, SS
	Reticular lines	IPF, asbestosis, chronic EP, chronic HP, CTD, NSIP
	Traction bronchiectasis	IPF, other end-stage fibrosis
	Honeycomb change	IPF, chronic EP and HP, asbestosis, sarcoidosis
	Ground-glass opacity	AIP, acute EP, PAP, chronic EP, COP, lymphoma, sarcoidosis, NSIP, infection, hemorrhage

AIP = acute interstitial pneumonia; AS = ankylosing spondylitis; CA = cancer; CBD = chronic beryllium diseases; COP = cryptogenic organizing pneumonia; CTD = connective tissue disease; DAH = diffuse alveolar hemorrhage; DM-PM = dermatopolymyositis; DIP = desquamative interstitial pneumonia; EP = eosinophilic pneumonia; HP = hypersensitivity pneumonitis; HPS = Hermansky-Pudlak syndrome; IPF = idiopathic pulmonary fibrosis; LAM = lymphangioleiomyomatosis; LCH = Langerhans cell histiocytosis; LIP = lymphoid interstitial pneumonia; NF = neurofibromatosis; NSIP = non-specific interstitial pneumonia; PAG = polyangiitis with granulomatosis; PAP = pulmonary alveolar proteinosis; P2 = auscultated pulmonic valve closure sound; PH = npulmonary hypertension; PVOD = pulmonary veno-occlusive disease; RA = rheumatoid arthritis; SLE = systemic lupus erythematosus; SS = Sjögren’s syndrome; TS = tuberous sclerosis.

Reprinted with permission from *Interstitial Lung Disease: A Practical Approach*. Meyer KC, Raghu G: **Patient evaluation**. Second Edition, New York: Springer; 2011:3–16.

**Figure 1:**
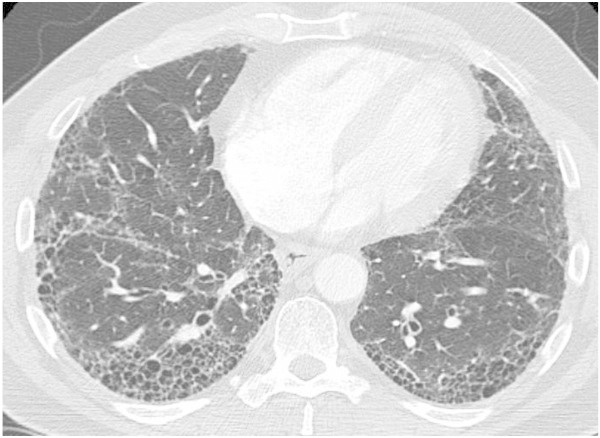
**HRCT cross-sectional view showing a pattern of peripheral reticulation and honeycomb change that is diagnostic of the presence of UIP.**

Although the combination of history, physical examination, CXR, and other appropriate laboratory testing (peripheral blood tests and lung physiologic testing) may provide a likely diagnosis, additional testing is usually needed to reach a confident diagnosis of a specific ILD. HRCT of the thorax can provide invaluable information that strongly supports a specific diagnosis (Table 5) and may be diagnostic (e.g. typical changes of UIP) such that further testing with bronchoscopy or surgical lung biopsy is not required (Figure 1). Indeed, the HRCT has become a standard test for the evaluation of patients with possible ILD [4]. In general, a complete lack of pulmonary parenchymal changes on HRCT imaging virtually excludes a diagnosis of ILD, although ILD may rarely still be present with the lungs having microscopic involvement that does not reach the threshold for the detection of an abnormality that is detectable by HRCT. Multi-detector computed tomography (MDCT) scanning can scan the entire thorax with a single breath-hold maneuver and allow even better imaging than traditional HRCT, and an algorithmic approach can be utilized that facilitates differentiation among UIP, NSIP, and chronic HP patterns [5].

### Invasive diagnostic procedures

**Figure 2:**
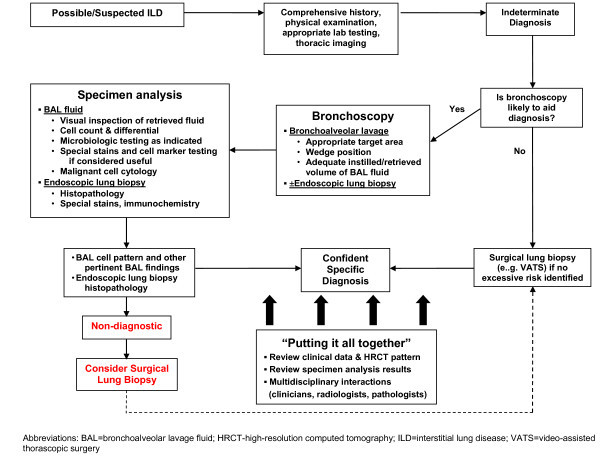
**Suggested approach to the diagnosis of ILD.** Abbreviations: BAL = bronchoalveolar lavage fluid; HRCT-high-resolution computed tomography; ILD = interstitial lung disease; VATS = video-assisted thorascopic surgery.

Bronchoscopy and/or surgical lung biopsy may be required to make a confident diagnosis of a specific ILD (Figure 2). Bronchoscopy is generally a very safe procedure if performed by an experienced bronchoscopist [6], and the most serious potential complications are pneumothorax or excessive bleeding that may occur as a consequence of transbronchial biopsy (TBLBx). Bronchoalveolar lavage (BAL) can be readily performed, and the recently published, ATS Task Force Report on BAL for the diagnosis of ILD recommends using recently obtained HRCT imaging to choose an appropriate segment of the lung in which to perform BAL from a wedge position [7]. The right middle lobe or lingula of the left upper lobe are likely the best regions to perform lavage when diffuse disease is present, and areas with ground-glass opacification or profuse nodular change are more likely to provide useful diagnostic information (e.g. differential cell count of nucleated immune cells) than areas with extensive fibrosis. In addition to total and differential BAL cell counts, BAL fluid and sediment can be analyzed for infection or the presence of malignant cells, and the gross appearance of freshly retrieved BAL fluid may provide diagnosistic information (e.g. progressively increasing blood in sequential aliquots that is seen with diffuse alveolar hemorrhage or white-tan discoloration of the BAL fluid with rapidly settling tan sediment [due to gravity] that can be seen with pulmonary alveolar proteinosis). Significant BAL lymphocytosis or eosinophilia may provide strong support for a specific diagnosis when combined with imaging and clinical data, but routine determination of BAL lymphocyte subsets is unlikely to provide additional useful information [7, 8].

Endobronchial biopsy may provide useful information if endobronchial abnormalities are present (e.g. superficial nodules, mucosal ulceration). Similar to performing BAL, TBLBx is best performed away from areas of advanced fibrosis (as identified by HRCT). Multiple biopsies performed with an adequately sized forceps can provide good tissue sampling for some forms of ILD (e.g. sarcoidosis), but TBLBx is likely to be non-diagnostic when extensive/advanced fibrotic disease is present.

A surgical lung biopsy (SLB) obtained via video-assisted thoracic surgery (VATS) or open biopsy is likely to provide an excellent specimen (if properly performed) that shows a histopathologic pattern that can usually be considered to be definitively diagnostic of a specific disease entity. However, one must weigh risks and benefits when performing a SLB is considered, especially in frail elderly patients, patients with ventilatory compromise, patients with moderate to severe pulmonary hypertension, or patients with multiple co-morbidities. Open lung biopsy in patients with suspected ILD has approximately a 4.3% 30-day mortality rate, whereas VATS biopsy appears to be safer with an associated 30-day mortality that is somewhat lower than open biopsy but not negligible at approximately 2.1% [9]. Additionally, the highest mortality risk may occur in patients whose ultimate diagnosis is IPF [10, 11], and SLB in patients with IPF has been reported to trigger an acute exacerbation of IPF [12]. However, confirming the diagnosis and differentiating among specific forms of IIP may not be possible without performing SLB.

There is increasing awareness that an abnormal degree of gastroesophageal reflux (GER) combined with aspiration may play a significant role in the pathogenesis of a number of forms of ILD [13, 14]. GER disease (GERD) has been associated with IPF and with pulmonary fibrosis in patients with systemic sclerosis (scleroderma), and GER with aspiration may play a role in triggering and/or driving lung inflammation and fibrosis in IPF and scleroderma, and it has been linked to acute exacerbations in patients with IPF [15, 16]. Additionally, esophageal dysmotility may play a role in predisposition to aspiration and is usually found in patients with scleroderma and may be present in patients with other forms of CTD with associated ILD [14, 17, 18]. Esophageal manometry to detect esophageal functional abnormalities plus impedance/pH testing to detect and characterize GER may provide evidence of foregut functional abnormalities that may play a role in IPF and possibly CTD-ILD pathogenesis, and obtaining such information by placing esophageal probes may provide findings that support the employment of strategies to prevent or blunt pathologic GER [19]. More research is needed in this area to better understand the role of GER and microaspiration in the pathogenesis of IPF and other forms of ILD.

### Making an accurate and confident diagnosis of a specific form of ILD

Patients with suspected ILD should have information from the history, physical examination, thoracic imaging, and other testing (e.g. peripheral blood tests, pulmonary function testing) carefully and thoughtfully reviewed to determine whether or not additional procedures are needed and whether such procedures are likely to be helpful in reaching a confident diagnosis. If one needs to obtain invasive testing (bronchoscopy with BAL and/or TBLBx, VATS biopsy), all findings should be reviewed (preferably in a multi-disciplinary fashion) to identify the ultimate diagnosis that best fits with the combination of clinical information, imaging, and invasive testing results [20]. Risks and potential benefit of invasive testing should be carefully considered, especially for frail, elderly patients.

Some specific combinations of clinical data with imaging results and other findings can strongly support specific ILD diagnoses. A younger patient with nodular changes along bronchovascular structures and bilateral hilar lymphadenopathy on HRCT imaging is highly likely to have a diagnosis of sarcoidosis, and the presence of a significant lymphocytosis on a BAL cell count determination would be highly supportive of this diagnosis. A patient with clearly abnormal autoimmune serologies and either a NSIP or UIP pattern on HRCT imaging is likely to have CTD-ILD and may have a specific CTD diagnosis such as rheumatoid arthritis or scleroderma that can be detected and confirmed via peripheral blood serologic testing. A patient with a significant exposure history to potential organic antigens (e.g. bird fancier or farmer) with a HRCT findings of upper lung field dominant centrilobular ground glass nodules, acute or subacute onset of symptoms (e.g. dyspnea, myalgias) is quite likely to have acute HP, and this diagnosis is strongly supported by the findings of significant BAL lymphocytosis. Similarly, a patient with such an exposure history plus a subacute or chronic symptom onset and a HRCT that shows ground glass opacities or fibrotic changes with extensive mosaic attenuation due to air-trapping is likely to have chronic HP. Lastly, an older patient who presents with subacute or chronic disease onset and has bibasilar Velcro crackles on chest auscultation is highly likely to have IPF as their specific ILD diagnosis, and this diagnosis can be confidently confirmed if the HRCT shows a typical UIP pattern (peripheral and basilar predominance of fibrotic changes with reticulation and honeycomb change (Figure 1) and very little or no ground glass opacities) and alternative etiologies are lacking (e.g. presence of CTD, asbestosis, drug reaction with fibrosis).

### Key management decisions

Once a confident diagnosis has been reached, a plan to treat and monitor disease activity can be put in place (Table 6). Key management decisions include whether to administer pharmacologic agents, how the disease will be monitored to determine whether it has stabilized or improved versus progressive deterioration, whether a patient should be referred for lung transplantation, and whether the disease is end-stage and unlikely to respond to therapies such that providing supportive, palliative care is the best approach. A treatment plan should not only consist of pharmacologic agents that are prescribed to prevent progression and/or induce remission (if the specific disorder can respond to such) but should also include supportive therapies (e.g. supplemental oxygen if indicated, pulmonary rehabilitation), measures to relieve symptoms (e.g. cough, anxiety, depression, dyspnea) and treatment of co-morbid conditions (e.g. anemia, sleep-disordered breathing, GER, infectious complications).

**Table 6:** **Management of the patient with ILD/IPF**

▪ Establish a partnership with the patient to provide a patient-centered, personalized medicine care plan	▪ Provide supplemental oxygen if indicated (keep SpO_2_ ≥90%)
▪ Provide patients with:	· During exertion
	· Nocturnal during sleep
• Useful information concerning the nature of their disease and its prognosis	· Continuous if indicated
• Treatment options accompanied by thoughtful counseling	▪ Detect and treat co-morbidities and complications:
· Enrollment in clinical trials	· Gastroesophageal reflux disease
· Off-label therapies (e.g. corticosteroids, cytoxic drugs, other agents)	· Cardiovascular disease
· Lung transplantation	· Drug toxicity (if treated)
· Best supportive care	· Sleep-disordered breathing
▪ Use disease-specific monitoring (for prognostication and treatment decisions)	· Secondary pulmonary hypertension
· Pulmonary function testing (FVC, DL_CO_, 6-MWT)	· Metabolic bone disease (osteopenia, osteoporosis)
· Thoracic imaging	· Anemia
· Dyspnea score	· Anxiety & depression
▪ Pulmonary rehabilitation (optimal exercise program, patient education)	▪ Maintain ideal body-mass index (weight reduction if obese, improved nutrition if cachectic)
	▪ Vaccinations (pneumococcal vaccine, seasonal influenza, others as indicated)

DLCO = diffusion capacity of the lung for CO; FVC = forced vital capacity; 6-MWT = six-minute walk test; SpO2 = oxyhemoglobin percent saturation.

Measurements that can be made periodically to objectively assess changes in physiologic function over time include formal dyspnea assessment tools, the forced vital capacity (FVC), diffusion capacity of the lung for carbon monoxide (DLCO), and the 6-minute walk test (6-MWT) distance and oxyhemoglobin saturation change [21–26]. The baseline FVC value has not been shown to correlate well with disease course for patients with IPF, but change in FVC over time has been show to correlate well with stable versus progressive disease with greater than 10% decline considered to be significant and indicative of disease progression [27, 28]. A decline of ≥15% in DLCO has also been correlated with disease progression in IPF [28], and declining 6-MWT distance or oxyhemoglobin saturation are also associated with disease progression [25, 29]. More recent analyses suggest that changes in FVC that are less than 10% may represent important clinical change [30], and using relative change rather than absolute change in FVC values may provide a better indication of clinical response [31]. In addition to its utility in diagnosis, HRCT can be scored for the extent/severity of fibrosis, and the fibrosis severity scoring has been shown to correlate with prognosis [32, 33]. However, the use of serial HRCT scanning has not been validated as a useful gauge of disease progression over time for IPF and presents a significant radiation risk to the patient. Biomarkers that reflect disease severity and correlate with prognosis have been reported for IPF [34, 35], but these have yet to be validated for use in the clinical setting.

Immunosuppressive anti-inflammatory agents are used to treat various forms of ILD (Table 7) [36, 37]. Although treatment of any form of ILD with immunosuppressive therapy is off-label in the U.S. and anti-inflammatory/immunosuppressive pharmacologic therapy has not been validated in placebo-controlled clinical trials, there is reasonably compelling evidence that the administration of agents such as corticosteroids is strongly associated with improvement or even clearing of lung pathology for many forms of ILD. This is particularly the case for disorders such as cryptogenic organizing pneumonia (COP), eosinophilic pneumonia, sarcoidosis, or cellular non-specific interstitial pneumonia (NSIP) [36].

**Table 7:** **Therapies for select types of ILD**

ILD type	Key features of immunopathogenesis	Current therapy*	Additional and/or alternative therapies
IPF	• Prominent fibroblast proliferation and matrix deposition	Supportive care	Anti-reflux therapy
	• Patchy, temporally heterogeneous changes	Consider anti-reflux measures	N-acetylcysteine
	• Architectural distortion of tissue	- Anti-reflux surgery	Clinical trials
	• Epithelial injury, microvascular remodeling	- Acid suppressants (e.g. PPI)	(experimental)
	• Variable inflammatory component (usually minimal/mild)	Pirfenidone (not approved in US)	
	• Areas of NSIP- and DIP-like change often present		
	• PH frequently present with advanced disease	Lung transplantation	
Sarcoidosis	• Well-formed non-caseating granulomata in tissues	Observation (mild/stable disease)	Infliximab
	• Extra-pulmonary disease may be present		Other IS agent
	• May be asymptomatic; may resolve spontaneously without therapy	Corticosteroids (oral or inhaled)	Lung transplantation
		Methotrexate	
NSIP	• Homogeneous, diffuse involvement of the lung	Corticosteroids	Other IS drugs
	• Histopathologic subtypes include cellular (prominent lymphocyte influx; best prognosis), mixed (cellular & fibrotic), & fibrotic (worst prognosis)	Mycophenolate	Lung transplantation
	• Usually responsive to IS (less likely to respond if advanced fibrosis is established)		
COP	• Prominent inflammatory cell infiltrate (↑ lymphocytes, neutrophils, and/or eosinophils can all be present)	Corticosteroids	Other IS drugs
			Macrolides
	• Usually responds to IS therapy; relapse frequently occurs		
HP	• Prominent lymphocyte influx with formation of loose granulomata	Exposure cessation	Other IS drugs
	• Can have appearance of cellular NSIP or OP	Corticosteroids	Lung transplantation
	• Can progress to advanced fibrosis (and masquerade as IPF or fibrotic NSIP)		
Eosinophilic pneumonia	• Prominent influx of eosinophils	Corticosteroids	Other IS drugs
	• Usually responsive to IS therapy		
CTD-ILD	• Lung histopathology can reveal NSIP (common), UIP (less common); other ILD (e.g. OP, DIP, RBILD – very uncommon)	Corticosteroids	Anti-reflux therapy
		Mycophenolate	Lung transplantation
	• PH often present (with or without ILD)	Other DMARD agent(s)	Treatment of PH
AIP/DAD	• Intense inflammation and alveolar damage	Corticosteroids	Cytotoxic drugs
	• Hyaline membrane formation		
	• Prominent neutrophil influx early		

*Therapies that are usually administered on the basis of expert opinion and clinical trial results; none have received US Food and Drug Administration approval for the indication of ILD/IPF (but pirfenidone is approved for treatment of IPF in some countries, and many DMARD agents are approved for treatment of CTD).

*Abbreviations*: *AIP* acute interstitial pneumonia, *COP* cryptogenic organizing pneumonia, *CTD-ILD* connective tissue disease-associated ILD, *DAD* diffuse alveolar damage, *DIP* desquamative interstitial pneumonia, *DMARD* disease-modifying anti-rheumatic drug, *HP* hypersensitivity pneumonitis, *IPF* idiopathic pulmonary fibrosis, *ILD* interstitial lung disease, *IS* immunosuppression, *NSIP* non-specific interstitial pneumonia, *OP* organizing pneumonia, *PH* pulmonary hypertension, *RBILD* respiratory bronchiolitis with interstitial lung disease.

When extensive fibrosis is present, such therapies may be less efficacious, especially for patients with IPF, for whom currently available immunosuppressive or anti-fibrotic therapies are not recommended [38]. However, some forms of CTD-associated ILD (NSIP or UIP pathologies) have been reported to respond to mycophenolate therapy, which also allowed a significant lowering of corticosteroid dosing [39]. If immunosuppressive agents are used to treat patients with ILD, treating clinicians should be adequately familiar with potential adverse reactions and drug-drug interactions, and guideline precautions (including recommended monitoring) should be followed [40]. Anti-fibrotic pharmacologic therapies are being increasingly brought to clinical trials [41, 42], and patients should be encouraged to enroll in clinical trials if they are found to have IPF or other forms of advanced ILD for which effective therapies have yet to be identified and clinical trials for their specific form of ILD are open to enrollment.

### Treatment of IPF

The prognosis of IPF is generally poor, and the majority of patients have progressive loss of lung function and may suffer acute exacerbations with acceleration of lung function loss that often leads to death [43, 44]. Traditional therapies that were suggested to benefit patients with IPF included corticosteroids and cytotoxic drugs (e.g. azathioprine, cyclophosphamide) [45]. However, these agents have never been shown to have significant benefit in any adequately powered, prospective, randomized, placebo-controlled clinical trial. Furthermore, it was recently demonstrated that azathioprine, an agent that has been suggested to have efficacy for the treatment of IPF [45–47], was associated with significant harm compared to placebo when administered to patients with IPF [48]. This observation triggered the termination of the azathioprine/N-acetylcysteine (NAC)/prednisone arm of the NIH-sponsored IPF PANTHER clinical trial when it became obvious that excess mortality and other complications occurred in this cohort versus the other study arms of either NAC alone or placebo. There are no treatment options for patients with IPF that have been approved by the U.S. Food and Drug Administration, and any pharmacologic treatment given in the US would be considered off-label.

Many new agents that target fibrogenesis have been evaluated in Phase 3 clinical trials (Table 8), but some of these agents (e.g. bosentan, macitentan, ambrisentan, interferons gamma and beta) have not shown benefit despite pre-clinical studies or phase 2 clinical trials that suggested potential efficacy. Indeed, there can be considerable inter-individual variability in genetic abnormalities that have predisposed an individual to develop the disease, in pathophysiologic characteristics of the disease process, and in responses to specific drugs. It should be recognized that a subset of patients that may benefit from a promising drug are very unlikely to be identified in a prospective, double-blind, randomized phase 3 clinical trial in which these patients are combined with a much larger number of enrolled subjects for whom the drug has little or no effect, and the conclusion may be reached that the drug lacks benefit despite its potential to help a subset of patients. Nonetheless, the results of some recently completed clinical trials suggest that pirfenidone [49, 50] or nintedanib (BIBF 1120) [51] may have a significant impact on disease progression versus placebo, and pirfenidone has been licensed and is clinically available in Japan, Europe, and Canada. Stem cell therapy, specifically the use of mesenchymal stem cells (MSC), has shown potential benefit in pre-clinical trials [52], and early results of a phase 1 clinical trial with adipose-derived MSC were recently reported [53].

**Table 8:** **Pharmacotherapy for IPF: agents in current clinical trials**

Agent	Target	Rationale
Pirfenidone	TGF-β, PDGF	Down-regulation of TGF-β-stimulated collagen synthesis and extracellular matrix accumulation and PDGF proliferative effects on fibroblasts
Tyrosine kinase inhibitors	Tyrosine kinase receptors	Inhibit fibrinogenic pathways by inhibiting receptor tyrosine kinase (RTK) binding by ligands (e.g. TGF-β, PDGF-B, CTGF, FGF, VEGF)
N-acetylcysteine	ROI (oxidant-antioxidant imbalance)	Replenish pulmonary glutathione stores and thereby antagonize signaling and tissue damaging effects of oxygen radicals (e.g. stimulatory effects of ROI on myofibroblasts)
Anti-TGF-β	TGF-β	Block TGF-β-induced fibroblast migration & proliferation, differentiation of myofibroblasts into fibroblasts, epithelial-mesenchymal transition, and resistance of myofibroblasts to apoptosis
Anti-CTGF	CTGF	Suppress fibroblast stimulation by CTGF
Anti-IL-13	IL-13	Inhibit induction of profibrotic cytokines (e.g. TGF-β, PDGF, IGF-1, PDGF, MMP-9)
Anti-LPA	Lysophosphatidic acid (LPA)	Prevent fibroblast recruitment into lung interstitium that can occur via the G protein-coupled LPA1 receptor
Anti-CCL2	CCL-2	Inhibit cell (e.g. lymphocytes, monocytes, fibrocytes) chemotaxis/influx to lung tissue and TGF-β expression
Anti-LOXL2	Lysyl oxidase-like protein-2 (LOXL-2)	Inhibit LOXL2-mediated fibroblast activation and deposition/accumulation of collagen
Plasma exchange, rituximab, steroids	Immune/inflammatory mediators	Suppress inflammation associated with an episode of acute exacerbation of IPF

*Abbreviations*: *TGF-β* transforming growth factor-β, *TNF-α* tumor necrosis factor-α, *ROI* reactive oxygen intermediates, *CTGF* connective tissue growth factor, *PH* pulmonary hypertension.

Comorbidities can have a significant impact on disease course and quality of life for patients with IPF and other fibrotic lung diseases [54, 55]. These include secondary pulmonary hypertension, coronary artery disease, venous thromboembolism, obstructive sleep apnea, coexistent emphysema, osteoporosis, diabetes mellitus, anxiety, and depression. Coronary artery disease is highly prevalent in patients with IPF [56, 57], and a significantly increased risk of developing primary lung cancer has been observed [58]. An increased risk of venous thromboembolism has also been observed [59], and sleep-disordered breathing is frequently present [60]. An IPFNet phase 3 clinical trial was performed to assess the effect of sildenafil in patients with idiopathic pulmonary fibrosis, but despite a trend toward improvement, a significant increase in 6MWT distance (the primary endpoint) was not attained [61], although a recent analysis of these data suggests that a subset of patients with right heart dysfunction may benefit from sildenafil therapy [62]. Similarly, anticoagulation, when given to disrupt the contribution of the coagulation cascade to the fibrotic process, provided no benefit and was associated with increased risk of significant adverse events [63].

An abnormal degree of GER, which is present in a majority of IPF patients and has been linked to the presence of pepsin and/or bile acids in BAL fluid [64], has also been considered to be an IPF-associated comorbidity. It has been suggested that reflux of foregut contents into the proximal esophagus via a dysfunctional lower esophageal sphincter (e.g. presence of a hiatal hernia) can predispose to (micro)aspiration, which may initiate and/or drive lung inflammation that can progress to pulmonary fibrosis in a susceptible individual, and accumulating evidence has linked GER with aspiration to IPF pathogenesis [13]. Use of medical therapy that inhibits acid production or having undergone a Nissen fundoplication has been associated with significantly improved survival for IPF patients [65], and an analysis of combined, placebo-arm cohorts from three IPFNet-sponsored studies has shown less FVC decline in subjects who were using acid-suppression therapy [16]. Additionally, high pepsin levels in BAL fluid have been linked to some cases of acute exacerbation of IPF [15], and a significantly reduced incidence of acute exacerbations of IPF was observed for subjects enrolled in combined placebo cohorts from the IPFNet phase 3 clinical trials if they were taking anti-reflux medication [66].

### Lung transplantation

Lung transplantation is an accepted form of treatment for patients with ILD that is progressive, clearly leading to respiratory failure, and refractory to other therapies [66, 67]. The number of lung transplants performed for patients with IPF surpassed that for COPD in 2007, when IPF became the leading indication for lung transplants performed in the United States [68]. Lung transplantation is the only form of therapy that may improve quality of life and survival for patients with IPF, and 5-year survival following lung transplantation for IPF or other forms of pulmonary fibrosis is approximately 50% [69].

Key decisions that pertain to a potential lung transplant recipient include timing of referral, whether criteria are met that allow a patient to be listed for the procedure, and whether to perform a single versus bilateral lung transplant. International Society for Heart and Lung Transplantation (ISHLT) guidelines [70] state that referral to a transplant center should be considered when the diagnosis of IPF or fibrotic NSIP is made due to the relatively poor prognosis for patients with fibrotic lung disease and, especially, for those with IPF. These guidelines also recommend that transplantation thresholds for patients with IPF include DLCO <40% predicted, >10% decline in FVC, or desaturation below 88% on pulse oximetry during a 6MWT. Potential candidates must be evaluated very carefully to detect issues that are contraindications to being allowed to proceed to the point of being placed on a lung transplant waitlist [71], and if a candidate is placed on a waitlist, the type of transplant that the candidate could potentially receive (e.g. single only, bilateral only, single or bilateral) must be determined. Single lung transplantation is a simpler operation that may be better tolerated by patients with ILD, and bilateral lung transplant has not been shown to have a better survival outcome than single lung transplant for patients with ILD (or the subset of patients with IPF) at our center [72]. Although lung transplant recipients are at considerable risk to develop a considerable number of complications [71, 73], patients can achieve good quality of life and enhanced survival following lung transplant [69].

### Future directions

Our understanding of the natural history and pathobiology of various forms of ILD continues to evolve, and classification systems, such as that for the IIPs [74], must be periodically revised to incorporate new knowledge. It is also clear from decades of research that the etiology and pathogenesis of IPF is highly complex [15, 75, 76] and likely involves an exposure stimulus (e.g. inhaled agents, aspirated gastrointestinal refluxate), genetic predisposition to consequent lung injury, and gene/genomics-directed aberrant repair responses that lead to sustained inflammation and matrix disruption/remodeling. Many clinical investigations with single agents have not shown benefit, and clinical trials with various agents have not simultaneously targeted multiple pathways (e.g. immune-mediated inflammatory responses plus abnormal myofibroblast behavior with progressive matrix deposition). Targeting only a single pathway or process rather than using a combination of agents may represent the “Achilles heel” of using monotherapy to treat IPF (and possibly other ILD with progressive fibrosis). While a single agent may have no significant impact on the clinical course of IPF, combination therapy may have an impact (e.g. combination therapy that includes an anti-fibrotic agent, immunomodulatory agent, anti-reflux therapy, and potent antioxidant). Selection of the appropriate endpoint measures for clinical trials may be key to identifying therapies that are clearly of benefit to patients with IPF [77, 78], and additional clinical research is much needed to identify effective therapies for IPF and other ILD associated with progressive fibrosis and early mortality. An improved understanding of the genetics and genomics of ILD will likely lead to the identification of new therapies that may have a significant treatment effect that relieves symptoms and restores quality of life for patients with significant, progressive ILD, but such therapies should have minimal risk of precipitating adverse reactions that can abrogate the benefits of pharmacotherapy.

## Conclusions

The diagnosis and treatment of the various types of ILD present a considerable challenge to clinicians. Nonetheless, a comprehensive clinical evaluation combined with appropriate imaging and diagnostic procedures can achieve a confident diagnosis of a specific type of ILD, and invasive testing with bronchoscopy or SLB may not be required. Treatment of ILD entities that are characterized by lung inflammation without the presence of extensive fibrosis can be quite successful when anti-inflammatory immunosuppressive agents are administered. However, when extensive fibrosis has become established, such therapies may have little or no impact on disease progression, especially in patients with IPF. Patients with progressive disease for which effective therapies are lacking should be encouraged to enroll in clinical trials if such are available, and lung transplantation can be considered for appropriate candidates. The diagnosis and treatment of comorbid conditions may also provide significant benefit to patients, and treating clinicians should focus on optimizing quality of life and symptom palliation for patients with advanced, progressive disease.

## Abbreviations

ATS: American thoracic society
BAL: Bronchoalveolar lavage
COPD: Chronic obstructive pulmonary disease
CT: Computed tomography
CTD: Connective tissue disease
CTD-ILD: Connective tissue disease-associated interstitial lung disease
CXR: Chest x-ray
DLCO: Diffusion capacity of the lung for carbon monoxide
FVC: Forced vital capacity
GER: Gastroesophageal reflux
GERD: Gastroesophageal reflux disease
HP: Hypersensitivity pneumonitis
HRCT: High-resolution computed tomography
IIP: Idiopathic interstitial pneumonia
ILD: Interstitial lung disease
IPF: Idiopathic pulmonary fibrosis
ISHLT: International society for heart and lung transplantation
MDCT: Multi-detector computed tomography
MSC: Mesenchymal stem cells
6-MWT: 6-minute walk test
NAC: N-acetylcysteine
NSIP: Non-specific interstitial pneumonia
SLB: Surgical lung biopsy
TBLBx: Transbronchial lung biopsy
UIP: Usual interstitial pneumonia
VATS: Video-assisted thorascopic surgery.
